# Evaluating the utility of two gestural discomfort evaluation methods

**DOI:** 10.1371/journal.pone.0176123

**Published:** 2017-04-19

**Authors:** Minseok Son, Jaemoon Jung, Woojin Park

**Affiliations:** Department of Industrial Engineering, Seoul National University, Seoul, South Korea; Tokai University, JAPAN

## Abstract

Evaluating physical discomfort of designed gestures is important for creating safe and usable gesture-based interaction systems; yet, gestural discomfort evaluation has not been extensively studied in HCI, and few evaluation methods seem currently available whose utility has been experimentally confirmed. To address this, this study empirically demonstrated the utility of the subjective rating method after a small number of gesture repetitions (a maximum of four repetitions) in evaluating designed gestures in terms of physical discomfort resulting from prolonged, repetitive gesture use. The subjective rating method has been widely used in previous gesture studies but without empirical evidence on its utility. This study also proposed a gesture discomfort evaluation method based on an existing ergonomics posture evaluation tool (Rapid Upper Limb Assessment) and demonstrated its utility in evaluating designed gestures in terms of physical discomfort resulting from prolonged, repetitive gesture use. Rapid Upper Limb Assessment is an ergonomics postural analysis tool that quantifies the work-related musculoskeletal disorders risks for manual tasks, and has been hypothesized to be capable of correctly determining discomfort resulting from prolonged, repetitive gesture use. The two methods were evaluated through comparisons against a baseline method involving discomfort rating after actual prolonged, repetitive gesture use. Correlation analyses indicated that both methods were in good agreement with the baseline. The methods proposed in this study seem useful for predicting discomfort resulting from prolonged, repetitive gesture use, and are expected to help interaction designers create safe and usable gesture-based interaction systems.

## Introduction

Gestures involving upper-extremity postures or motions (hereafter, simply UE gestures or gestures) are among the most basic means of human communication, along with speech and facial expressions [1]. People use gestures to complement and supplement speech. Also, gesture can stand on its own to substitute for speech [2,3]—the deaf communities indeed use sign languages composed of gestures for everyday communication.

As gesture recognition technologies mature and become more available, designed UE gestures are being increasingly utilized for human-machine interaction (HMI) [4–6]. Currently, applications of gesture-based interaction include machine/device control [7,8], data exploration in virtual reality [9], artistic creation [10], musical instrument playing [11], and games [12–15]. Gestures are also being utilized to evoke functions and commands during routine computer tasks, such as word processing [16] and web browsing [17–20], partly replacing the traditional keyboard and mouse-based input system. When working with many of these applications, human user may need to perform gestures repetitively over a long duration.

Prolonged, repetitive use of UE gestures can give rise to physical discomfort especially when the designed gestures are awkward and stressful. Excessive physical discomfort can adversely affect work productivity [21] and deteriorate user experience. It is also known to be associated with increased risks of musculoskeletal disorders [22–27]. Multiple previous research studies have reported that sign language interpreters, who use UE gestures extensively, experience musculoskeletal discomfort and pain, and, are at an increased risk of upper-extremity musculoskeletal disorders [28–31]. The design of gestures for HMI must accomplish controlling the physical discomfort associated with their use by identifying and excluding stressful design alternatives. Such discomfort control would be a necessary condition for wide acceptance of gesture-based interaction.

An ability to adequately evaluate the discomfort levels of different gesture design alternatives is required as a prerequisite to achieving physical discomfort control in the design of gestures. Especially, a means for characterizing and classifying gestures in terms of level of discomfort resulting from prolonged and repetitive gesture use, which is the operating condition of interest, is needed. Despite the need, however, gestural discomfort evaluation has not been extensively studied, and few methods with proven utility seem currently available.

Several previous studies on gesture vocabulary design utilized subjective rating to evaluate discomfort levels of designed gestures [32–42]. These studies did not precisely delineate the subjective rating protocol, and none of the previous studies clearly specified the details including the pace of gesture execution and the number of repetitive gesture executions needed prior to subjective rating. It is suspected that without clear instructions, the human evaluators in these studies performed each gesture once or repeated it a few times and determined its discomfort level based on the brief sensory experience—the authors observed that most people repeated the gesture four times or less when asked to evaluate its discomfort level without any particular instructions. Also, the previous studies adopting subjective rating did not clearly specify the type of discomfort to be evaluated, and, thus, left some ambiguity. The human evaluators may perform the subjective rating under either of two instructions: to express the degree of perceived discomfort directly resulting from the small number of gesture executions or to express the gesture’s discomfort level in association with some hypothetical long-term gesture usage scenario (e.g., evoking a certain frequently used function or command of a word processing software program used on a daily basis). The latter would be significantly more difficult than the former since it requires mentally extrapolating the sensation from a brief physical experience to a hypothetical situation; also, the latter would introduce variability in the discomfort scores due to inter-individual differences in the interpretation of the hypothetical long-term gesture usage situation.

Despite its use in multiple previous studies and the advantage that the evaluation can be rapidly conducted, whether or not subjective rating based on a few gesture executions is an adequate method is not clear. A small number of gesture executions would not create significant physical burden and the resulting sensory information may or may not be sufficient for the human evaluator to characterize and differentiate discomfort levels of gestures in association with the actual use condition of interest, that is, prolonged and repetitive gesture use.

One possible solution for problems related to gestural discomfort evaluation would be to utilize manual work assessment tools developed in the field of physical ergonomics, such as rapid upper limb assessment (RULA) [43], rapid entire body assessment (REBA) [44], assessment technique for postural loading on the upper body (LUBA) [45] and concise exposure index (OCRA) [46], Strain Index [47] and ACGIH Hand Activity TLV [48]. While these assessment tools were originally developed for analyzing industrial work tasks, they may well be useful for characterizing discomfort levels of UE gestures. This conjecture is based on the assumption that characteristics of uncomfortable UE gestures are not significantly different from those of stressful upper-extremity postures or motion patterns found in industrial tasks. The results from [31], which identified gesture features related to hand and arm pain and fatigue, lend support to this view. The utilization of existing ergonomics work assessment tools seems to provide some advantages, including that: 1) their utilities in quantifying the risks of work-related musculoskeletal disorders have been confirmed repeatedly in the fields, and 2) they can minimize costly and time-consuming empirical data collection. Despite their potential utilities, however, the utilization of ergonomics manual work assessment tools has not been explored in the context of gestural discomfort evaluation, and its effectiveness in quantifying discomfort resulting from prolonged, repetitive gesture use remains to be demonstrated.

The lack of gestural discomfort evaluation method with proven utility is problematic as it hampers designing safe and usable gesture-based interaction systems. As an initial effort towards addressing this problem, the objective of the current study was to evaluate two gestural discomfort evaluation methods in their capabilities to predict discomfort resulting from prolonged, repetitive gesture use. One of the two methods was a subjective rating method termed the quick rating (QRating) method, and the other was a method of utilizing the existing RULA posture analysis tool termed the modified RULA (M-RULA) method [43]. To eliminate the uncertainty in the rating protocol mentioned earlier, the QRating method specifies that the rater performs the subjective rating with a maximum of four gesture repetitions and also express the discomfort directly resulting from the gestural repetitions rather than extrapolating it to some hypothetical long-term gesture usage scenario. The M-RULA method considers a gesture as a set of postures and analyzes each posture using a modified version of RULA. RULA quantifies the risks of work-related musculoskeletal disorders for industrial manual tasks according to a predetermined posture classification and scoring scheme. The two methods were evaluated through comparisons against a baseline discomfort evaluation method involving actual prolonged, repetitive gesture use. The baseline evaluation method quantifies the average rate of increase in perceived discomfort during prolonged, repetitive use of a gesture, and, thus, was termed the discomfort increase rate (IncreaseRate) method. The IncreaseRate method involves human evaluators actually performing a gesture repeatedly over a long duration until the perceived discomfort reaches a predetermined, significant level. The IncreaseRate method is considered as providing a baseline for evaluating the QRating and the M-RULA methods as it simulates prolonged, repetitive use of a gesture, which is the operating condition of interest.

## Gestural discomfort evaluation methods

This section describes in detail the two gestural discomfort evaluation methods examined in this study, the QRating method and the M-RULA method.

### 2.1 Quick Rating (QRating) method

**Fig 1:**
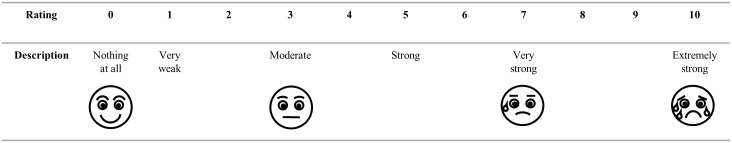
A 10-point scale for evaluating gestural discomfort.

The QRating method requires a group of human evaluators (user representatives) to perform a gesture a few times (a maximum of four repetitions) and subjectively rate the discomfort level of the gesture. We limited the number of repetitions to four on the basis of our observations in reality—we consistently observed that when asked to evaluate a gesture’s discomfort level, without any particular instructions, most people repeated the gesture four times or less. This was observed across many individuals we saw and also for different gestures. The group mean of the subjective ratings was used as the gestural discomfort measure. The participants were instructed to quantify the level of discomfort directly resulting from the repetitions of the gesture rather than extrapolating it to some hypothetical long-term usage scenario. This was because such mental extrapolation would be difficult to perform and would introduce variability due to individual differences in the interpretation of hypothetical long-term usage scenario. Subjective rating involves the use of a 10-point modified Borg scale [49] (Fig 1).

### 2.2 The RULA-based gestural discomfort evaluation method

This section describes the existing RULA and the M-RULA examined in this study. The M-RULA method follows the original computation scheme of RULA, but is slightly modified for the purpose of gesture evaluation.

#### 2.2.1 Rapid Upper Limb Assessment (RULA)

RULA is an ergonomics posture analysis tool [43] and is widely used to evaluate and redesign various industrial manual work tasks [50–53]. When given a work task, RULA produces a numerical score representing its physical stress level. The RULA score has shown good correlation with self-reported musculoskeletal discomfort and pain related to occupational work tasks [53–55] and is considered useful in assessing the risks of work-related musculoskeletal disorders [56–60]. Its repeatability and reliability have also been demonstrated [43].

**Fig 2:**
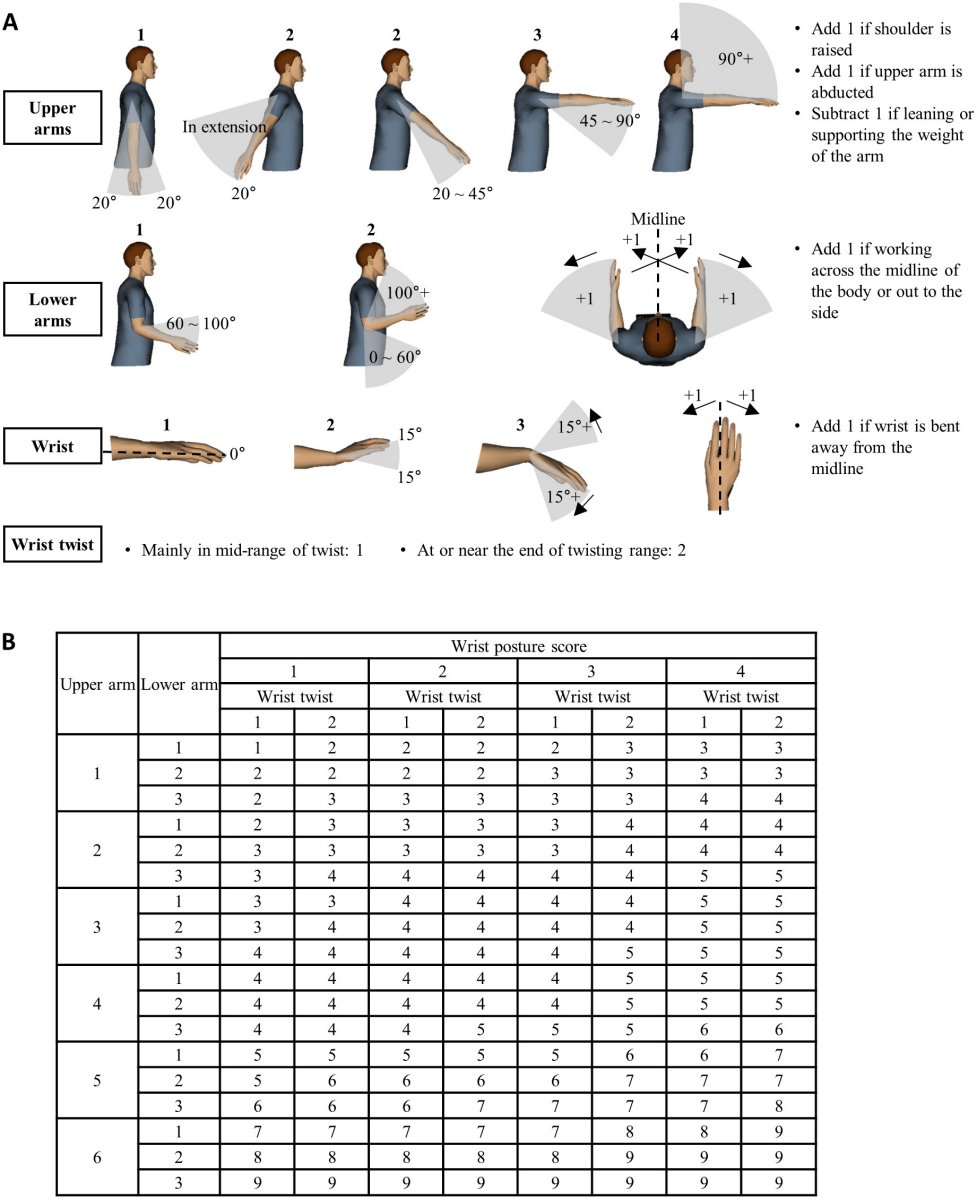
The RULA scoring system for Group A (the upper arm, lower arm and wrist). (A) The body part posture classification and scoring schemes for Group A. (B) The lookup table for determining the overall score for Group A.

To determine the score for a given work task, RULA uses a predetermined posture classification and scoring system. The posture classification and scoring system of RULA divides the human body into two groups, Group A and Group B [43]. Group A consists of upper arms, lower arms and wrists, whereas Group B includes neck, trunk and legs. For each group, the corresponding body parts are analyzed individually. For each body part, its range of movement is divided into a few pre-determined joint angle intervals. Each interval has a numerical score representing the level of physical stress it imposes on the body part. Fig 2A shows the body part posture classification and scoring schemes for Group A. In analyzing a whole-body posture with RULA, a human analyst observes each body part’s position and finds the interval that it belongs to; then, the corresponding body part posture score is determined. The posture scores determined for both groups of body parts are combined to produce a single overall score. Fig 2B presents the lookup table for determining the overall score for Group A. Finally, the overall scores of Groups A and B are combined to produce the grand score for the whole body.

#### 2.2.2 Modified RULA (M-RULA) method

The M-RULA method is aimed at quantifying discomfort levels of UE gestures on the basis of RULA. While RULA was originally developed and has been used primarily for analyzing industrial manual work tasks, it has also been utilized for analyzing non-industrial tasks [61–63]. In this study, it was hypothesized that RULA can be used to evaluate gestures in terms of discomfort resulting from prolonged, repetitive gestural interaction. This hypothesis was predicated upon the assumption that uncomfortable UE gestures would share characteristics with stressful industrial postures or motions. [31] lends support to this view—the study identified gestural features associated with hand and arm pain and fatigue, and found that they were largely identical to the characteristics of stressful industrial postures and motions reported in the ergonomics literature.

**Fig 3:**
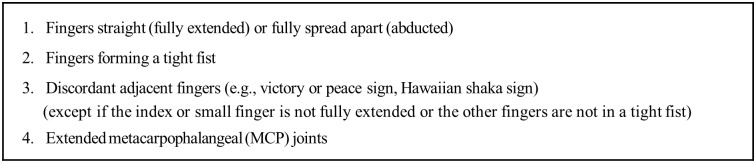
Four of the distinct characteristics associated with uncomfortable finger postures (Rempel et al., 2014).

The M-RULA method modifies the original RULA in two ways so as to quantify discomforts of UE gestures. First, as RULA does not consider thumb and finger postures in quantifying gestural discomfort, the M-RULA method incorporates the existing ergonomics knowledge on the discomfort of different thumb and finger postures into the discomfort score quantification scheme of RULA by adding extra points when applicable. Some distinct characteristics associated with uncomfortable finger postures identified in [31] are shown in Fig 3. The posture score (Group A score) is increased by 1 if the posture exhibits any one characteristic in Fig 3, and by 2 if the posture is accompanied with more than one characteristic. This “adding extra points” scheme is similar to the original scoring system of RULA, which adds muscle use and force scores (1 to 3 points) to the Group A score if necessary. Second, the M-RULA method deals with a gesture, which is a dynamic motion consisting of a continuous sequence of postures. To quantify the discomfort level of a gesture, the M-RULA method examines multiple postures sampled from a video clip of the gesture—typically, the two terminal (initial and final) postures and several postures in between for a discrete UE gesture. A discomfort score is determined for each sampled posture using the modified RULA scoring system. The highest discomfort score is chosen as the final M-RULA score representing the discomfort level of the gesture.

For a given UE gesture, the M-RULA method produces two gestural discomfort measures: the upper extremity (UE) and whole-body (WB) M-RULA scores. The UE M-RULA score corresponds to the Group A score and quantifies the level of upper extremity discomfort. The WB M-RULA score corresponds to the RULA’s grand score and quantifies whole-body discomfort.

The possible range of the UE M-RULA score is 1 through 11- an increase in the UE M-RULA score indicates increased risks of upper extremity musculoskeletal problems and increased physical discomfort in the context of prolonged, repetitive gesture use. The possible range of the WB M-RULA score is 1 through 7. The procedures for determining the WB M-RULA score (the RULA grand score) is described in [43]. The WB M-RULA score allows determining the requirements for action to eliminate risks to the operator (the user). The study defined four Action levels (Action levels 1~4) with each level corresponding to a specific RULA grand score [43].

## Evaluating the utility of the gestural discomfort evaluation methods

### 3.1 The baseline method

**Fig 4:**
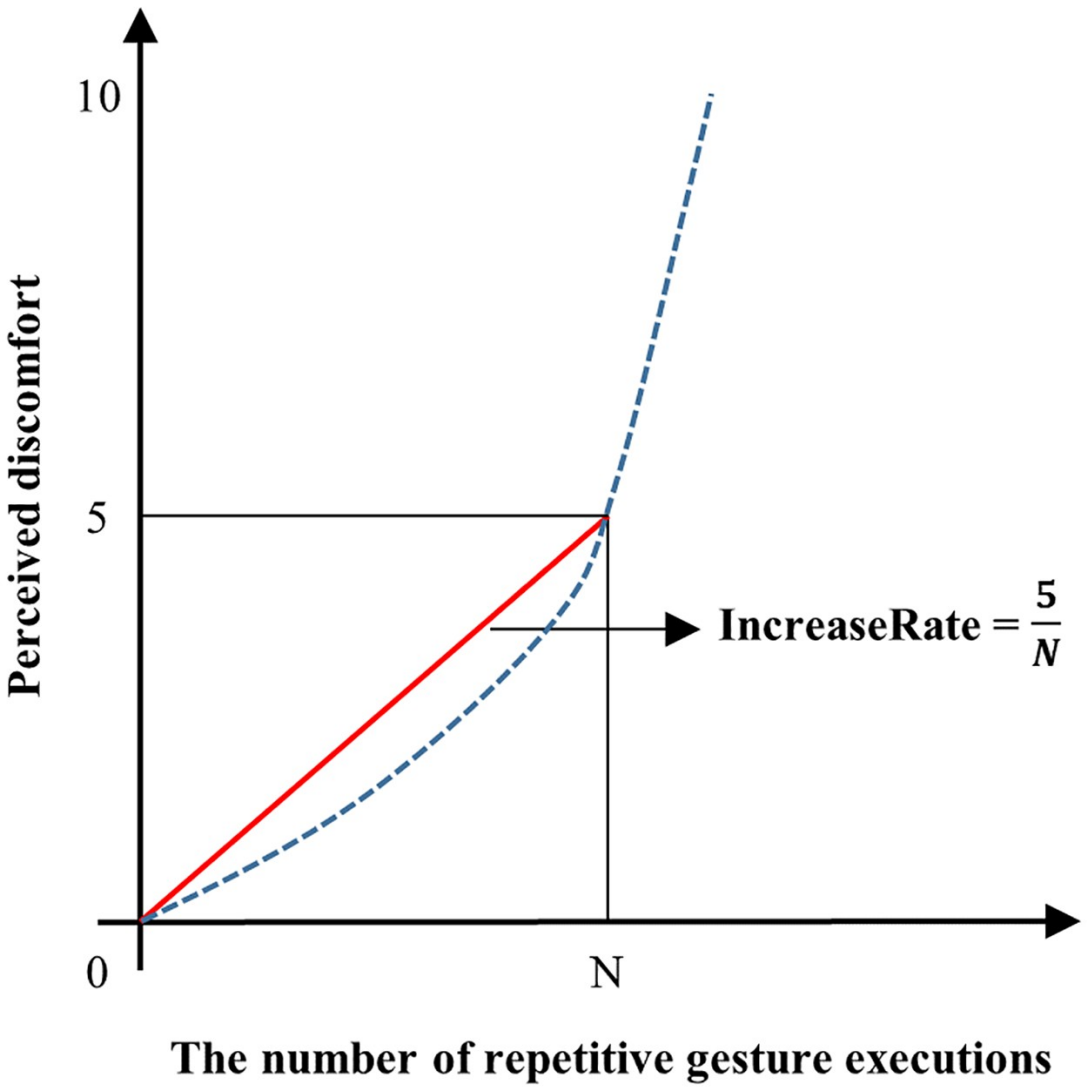
Discomfort increase rate (IncreaseRate).

The method of discomfort increase rate (the IncreaseRate method) was devised as a baseline for evaluating the utility of the QRating and the M-RULA methods. The IncreaseRate method quantifies a gesture’s discomfort level in terms of the average rate of increase in perceived discomfort (increase in discomfort per gesture execution) during continuous repetition of the gesture. For determining the IncreaseRate of a given gesture, a human subject continuously repeats the gesture at a natural, self-selected pace until the perceived discomfort reaches a predetermined, significant discomfort level. This study selected 5 (“strong”) on the 10-point Borg CR10 scale (Fig 1) as the predetermined discomfort level. The IncreaseRate is computed as the predetermined discomfort level divided by the number of repetitive gesture executions before reaching the level (Fig 4). For a given gesture to be evaluated, the IncreaseRate values are empirically determined for a sample of individuals, representative of the user population; then, the sample mean or a similar measure of central tendency is used as the discomfort measure. A more uncomfortable gesture results in a larger mean IncreaseRate than a less uncomfortable one.

The IncreaseRate measure is conceptually related to the notion of maximum holding time (MHT), which has been employed to empirically determine the physical stress levels of static working postures [22,45,64,65]. For a UE gesture with a low or medium discomfort level, a IncreaseRate evaluation session can result in a large number of gesture repetitions over a long duration. Since the IncreaseRate method quantifies gestural discomfort based on human perception after prolonged, repetitive gesture use, which is similar to the use condition of interest, it is served as a baseline for evaluating the QRating and the M-RULA methods.

### 3.2 Data collection and analyses

#### 3.2.1 Selecting a set of UE gestures

**Fig 5:**
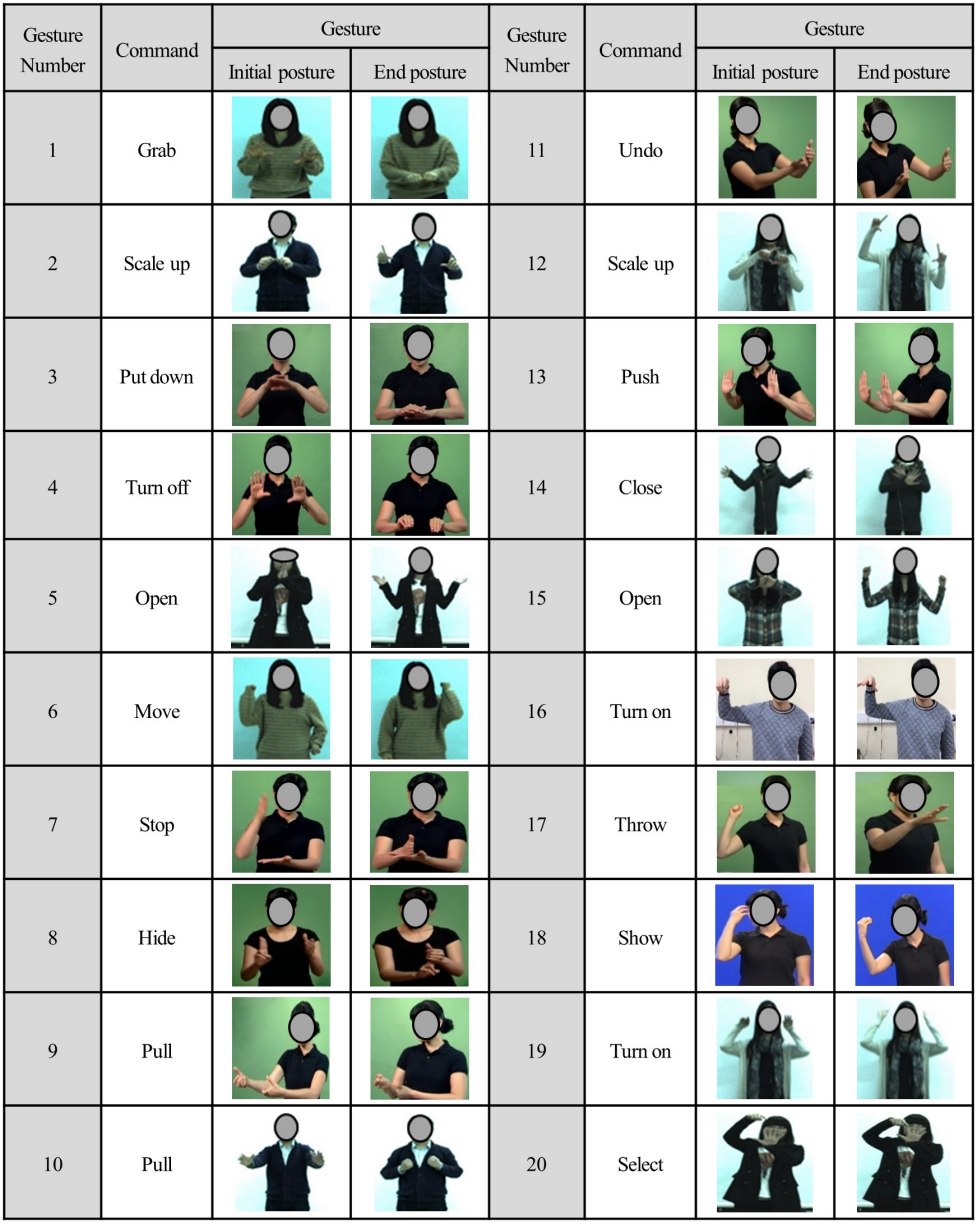
The twenty 3D UE gestures.

In a previous study conducted by one of the authors [66], UE gestures for different types of object manipulation in a virtual reality world were developed. The twenty gestures selected from the gesture set are described in Fig 5 –for each gesture, the initial and the final postures are provided. All of the twenty gestures selected were dynamic gestures involving UE movements.

#### 3.2.2 Evaluating discomfort levels of UE gestures using the QRating, the M-RULA and the IncreaseRate (baseline) methods

The discomfort levels of the twenty gestures were evaluated using the three different gestural discomfort evaluation methods—the QRating, the M-RULA, and the baseline (IncreaseRate) methods. For the QRating measure, sixteen participants rated the discomfort level of each gesture after repeating it a few times (a maximum of four times), using the 10-point scale shown in Fig 1. The order of the presentation of the twenty gestures was randomized for each subject. For each gesture, the average QRating scores of the participants was utilized for the subsequent data analyses. As for the M-RULA measure, for each gesture, the authors computed its UE and WB M-RULA scores using the scoring scheme described in section 2.2. Two video cameras (frontal and lateral) were used to supply video clips of each gesture. The Action level corresponding to each gesture was determined on the basis of its WB M-RULA score and the criterion described in [43].

For the baseline IncreaseRate measure, ten participants repeated each of the twenty gestures at a natural, self-selected pace, and for each gesture, the IncreaseRate values of the participants were determined. It is worth noting that the IncreaseRate and QRating measures were determined using different samples of people. The reliability of the QRating measure can be assured if it is in good agreement with the IncreaseRate measure even with the different sample used [67]. Each IncreaseRate session was video recorded, which allowed estimating the average duty cycle for each of the gestures. The duty cycle was defined as the time taken for one complete gesture execution, starting from and ending at the “ready” position (both hands down on one’s sides while standing erect). The presentation order of the twenty gestures was randomized for each subject. For each participant, the IncreaseRate sessions were conducted over two days (ten gestures per day) to minimize the influence of cumulative fatigue resulting from repetitive gesturing. For each subject, at least half an hour of rest was provided between consecutive trials of repetitive gesturing (on average, the total amount of experiment time for both the experiment trials and the rests was about 5 hours for each experiment day). In a pilot study prior to the experiment, half an hour of rest was found to be long enough to minimize the risk of cumulative fatigue effects. For each gesture, the average IncreaseRate values of the ten participants was utilized for the subsequent data analyses. The data collection protocol had been approved prior to the experiment by the Institutional Review Board of Seoul National University.

#### 3.2.3 Data analyses

For each gestural discomfort measure considered (the QRating, the UE M-RULA, the WB M-RULA and the IncreaseRate), the scores of the twenty gestures and their rank order were graphically visualized. The Spearman rank correlation analyses were conducted to examine the level of agreement between each of the gestural discomfort measures and the baseline measure. Also, the agreement between the QRating and each of the two M-RULA scores (the UE and the WB M-RULA scores) was also examined using the Spearman rank correlation analysis.

The statistical analysis package SPSS 20.0 for Windows was used for the statistical analyses and the α-level was set at 0.05.

### 3.3 Results

#### 3.3.1 Gestural discomfort

**Fig 6:**
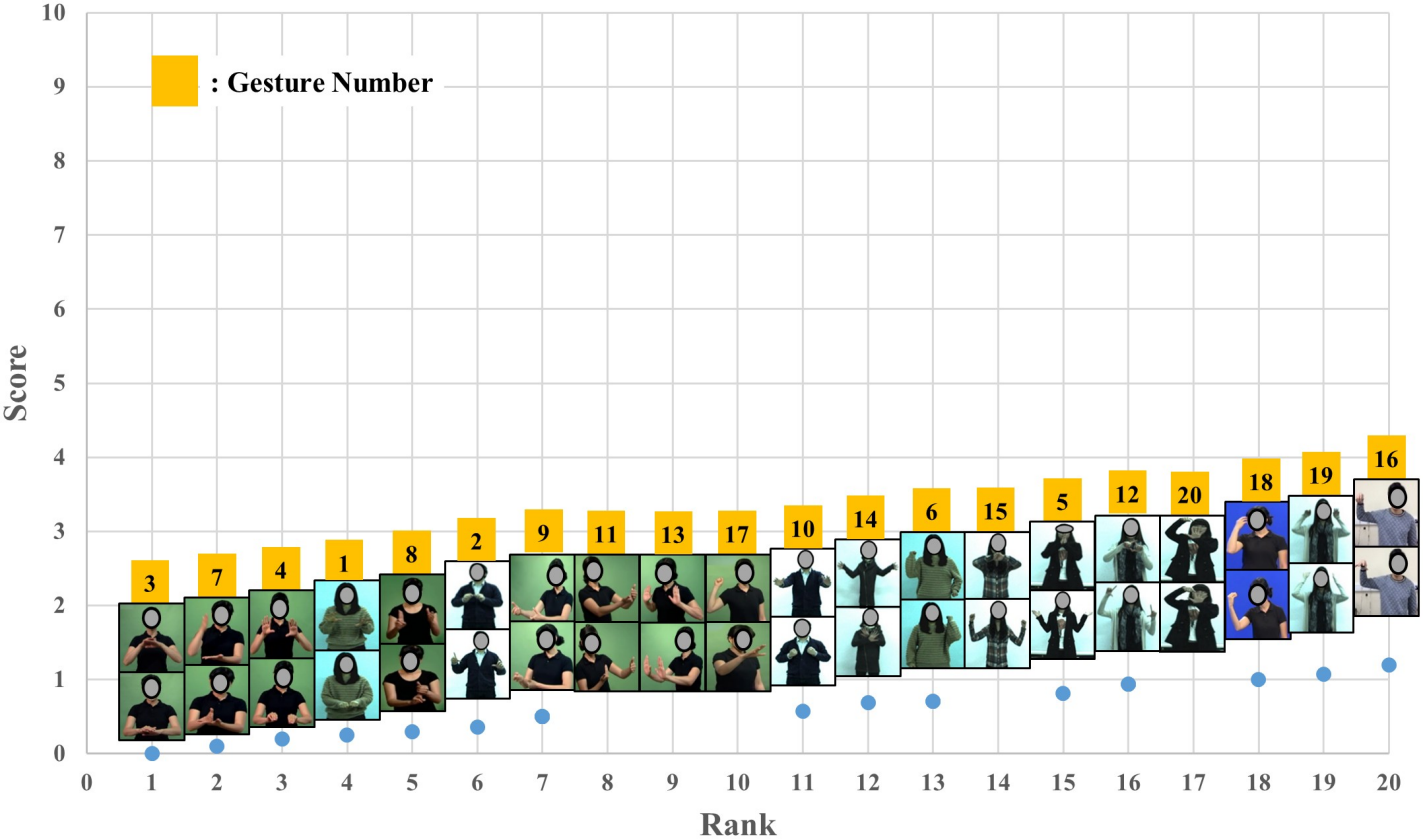
Mean QRating scores of the twenty gestures.

**Fig 7:**
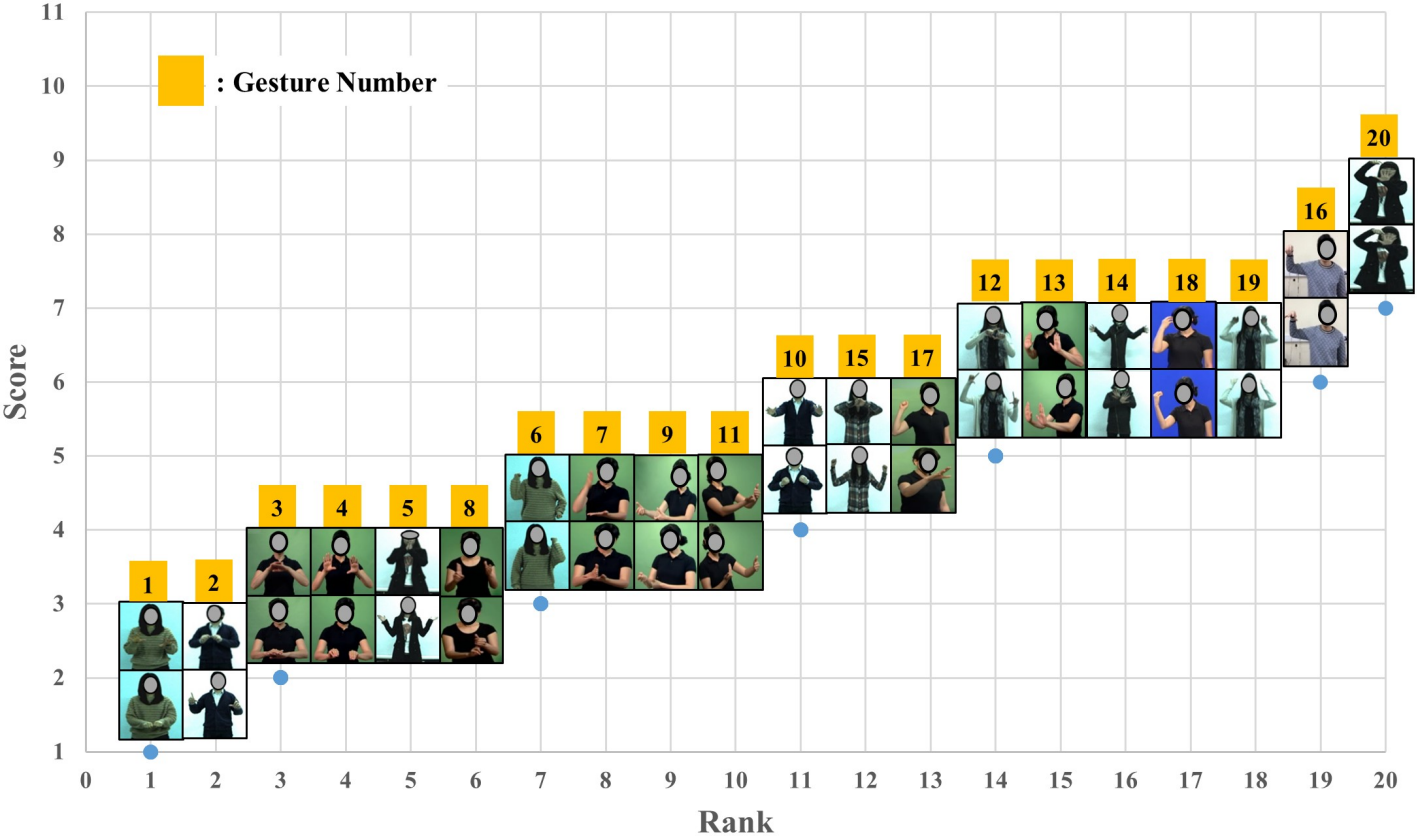
UE M-RULA scores of the twenty gestures.

**Fig 8:**
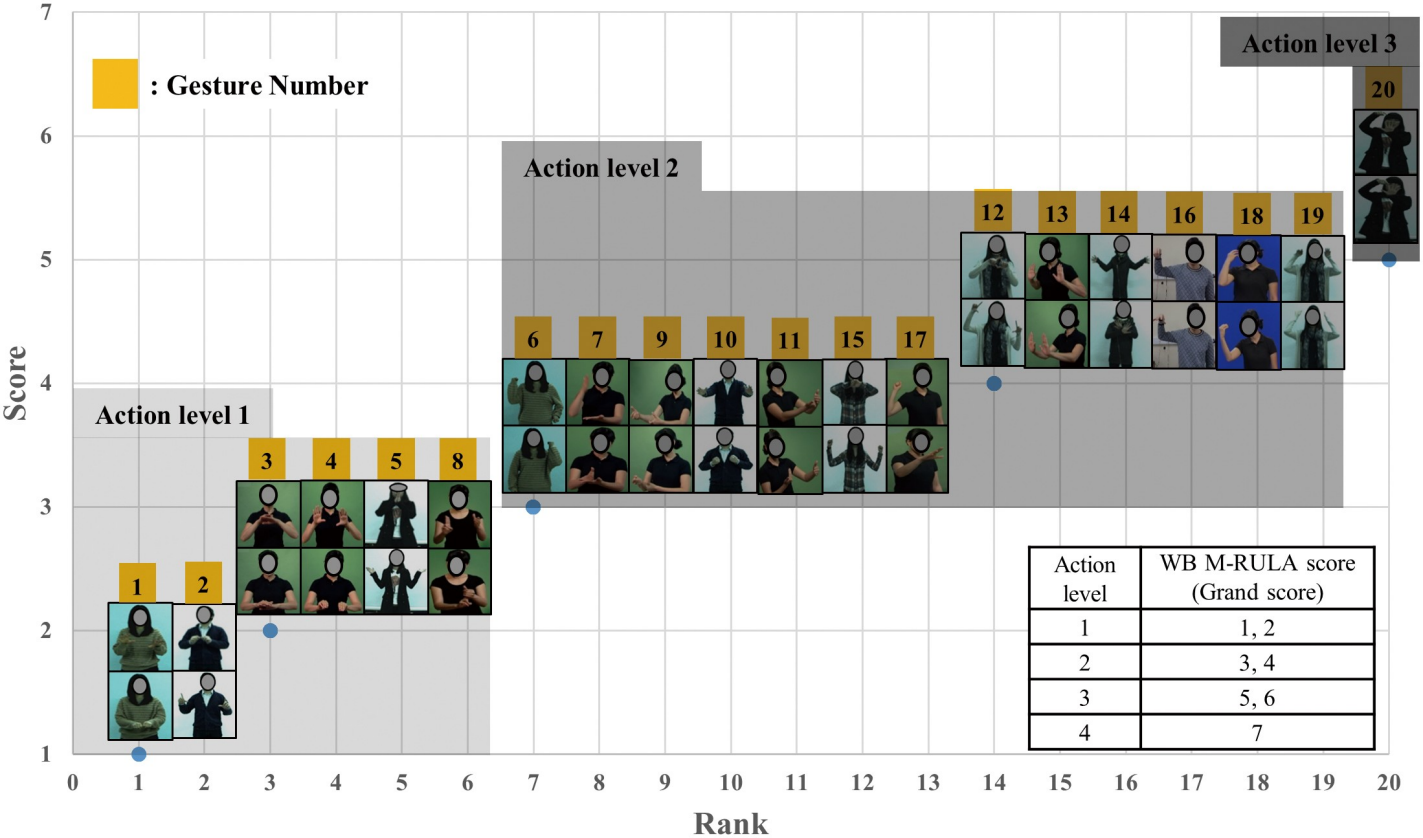
WB M-RULA scores of the twenty gestures.

**Fig 9:**
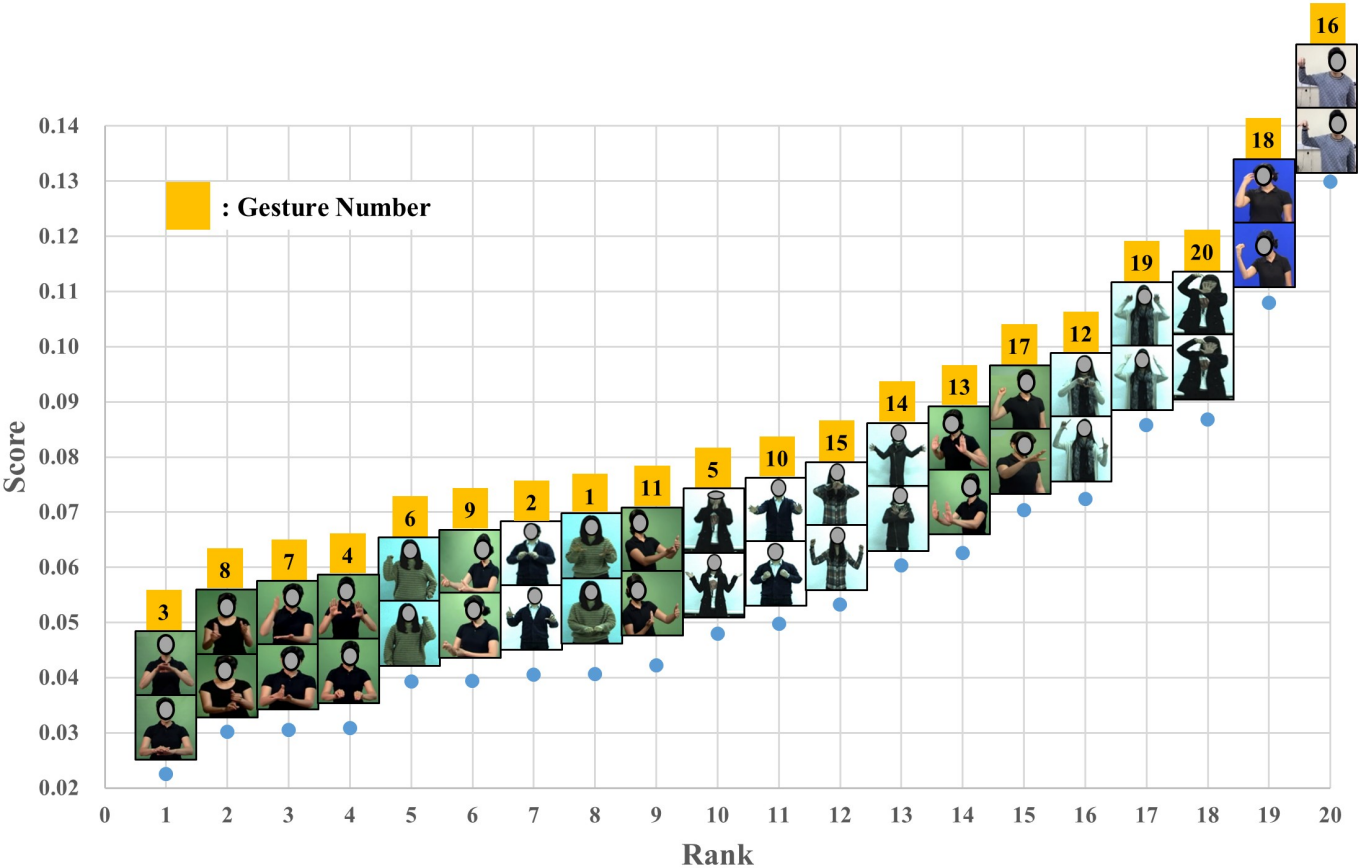
Mean IncreaseRate scores of the twenty gestures.

The mean QRating, the UE M-RULA, the WB M-RULA, and the mean IncreaseRate scores determined for each of the twenty gestures are provided in Figs 6–9 –each Fig shows the rank order of the twenty gestures for the corresponding measure. The WB M-RULA scores of the twenty gestures are accompanied with the corresponding RULA Action levels in Fig 8. Related to the baseline IncreaseRate measure, Table 1 provides the mean IncreaseRate score, the number of gesture repetitions and the duty cycle for each of the twenty gestures. With the target discomfort level of 5 on a 10-point scale, the participants in the current study repeated the gestures 107 times on average (the range was from 39 to 222 times). As for the within day order effect on the IncreaseRate scores, an ANOVA revealed that there was no significant effect of the presentation order (p-value = 0.880).

**Table 1:** The mean IncreaseRate score, the number of gesture repetitions and the duty cycle for each of the twenty gestures.

Gesture	Mean IncreaseRate score (SD)	Mean number of repetitive gesture executions (SD)	Mean duty cycle (SD) (in seconds)
1	0.041 (0.029)	123.1 (73.8)	1.88 (0.11)
2	0.041 (0.033)	123.4 (101.9)	1.92 (0.10)
3	0.023 (0.043)	221.9 (175.3)	1.92 (0.13)
4	0.031 (0.035)	162.0 (136.8)	1.91 (0.12)
5	0.048 (0.074)	104.3 (73.2)	2.31 (0.23)
6	0.039 (0.046)	127.0 (99.0)	2.18 (0.20)
7	0.031 (0.047)	163.6 (104.4)	1.99 (0.13)
8	0.030 (0.035)	165.8 (85.8)	1.95 (0.20)
9	0.039 (0.058)	126.8 (63.1)	1.88 (0.12)
10	0.050 (0.062)	100.5 (79.6)	2.05 (0.13)
11	0.042 (0.041)	118.5 (57.9)	2.12 (0.12)
12	0.072 (0.082)	69.1 (41.6)	2.27 (0.22)
13	0.063 (0.074)	79.8 (51.3)	2.09 (0.15)
14	0.060 (0.054)	82.9 (53.1)	2.27 (0.17)
15	0.053 (0.048)	93.9 (91.4)	2.25 (0.15)
16	0.130 (0.060)	38.5 (16.2)	2.32 (0.21)
17	0.070 (0.041)	71.0 (37.8)	2.26 (0.23)
18	0.108 (0.129)	46.3 (27.0)	2.19 (0.16)
19	0.086 (0.074)	58.3 (41.7)	2.42 (0.20)
20	0.087 (0.047)	57.6 (27.7)	2.58 (0.16)

#### 3.3.2 Spearman rank correlation coefficients

**Fig 10:**
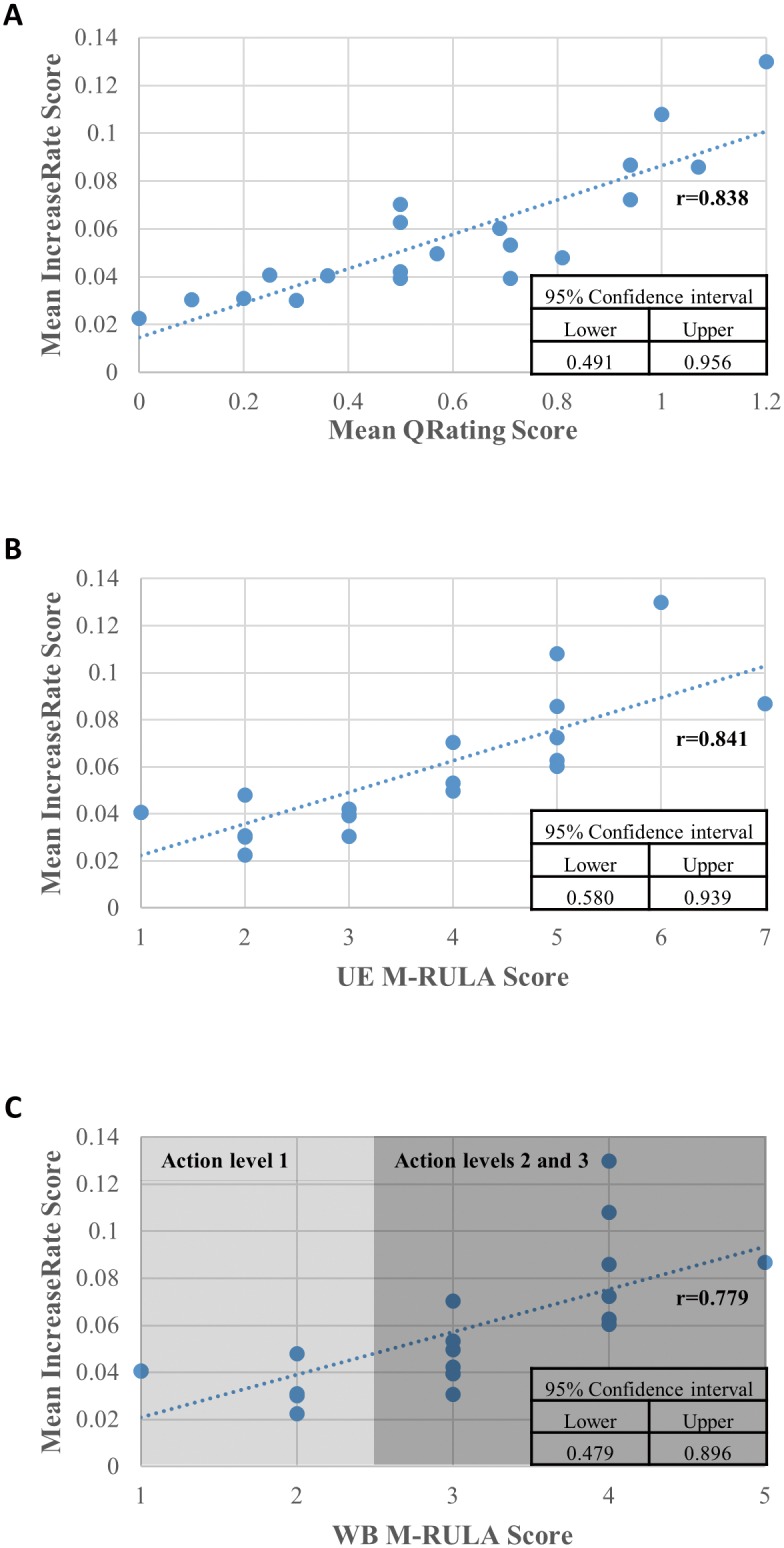
Relationship between each gestural discomfort measure and the baseline (IncreaseRate). (A) Relationship between mean IncreaseRate and QRating scores. (B) Relationship between mean IncreaseRate and UE M-RULA scores. (C) Relationship between mean IncreaseRate and WB M-RULA scores.

**Fig 11:**
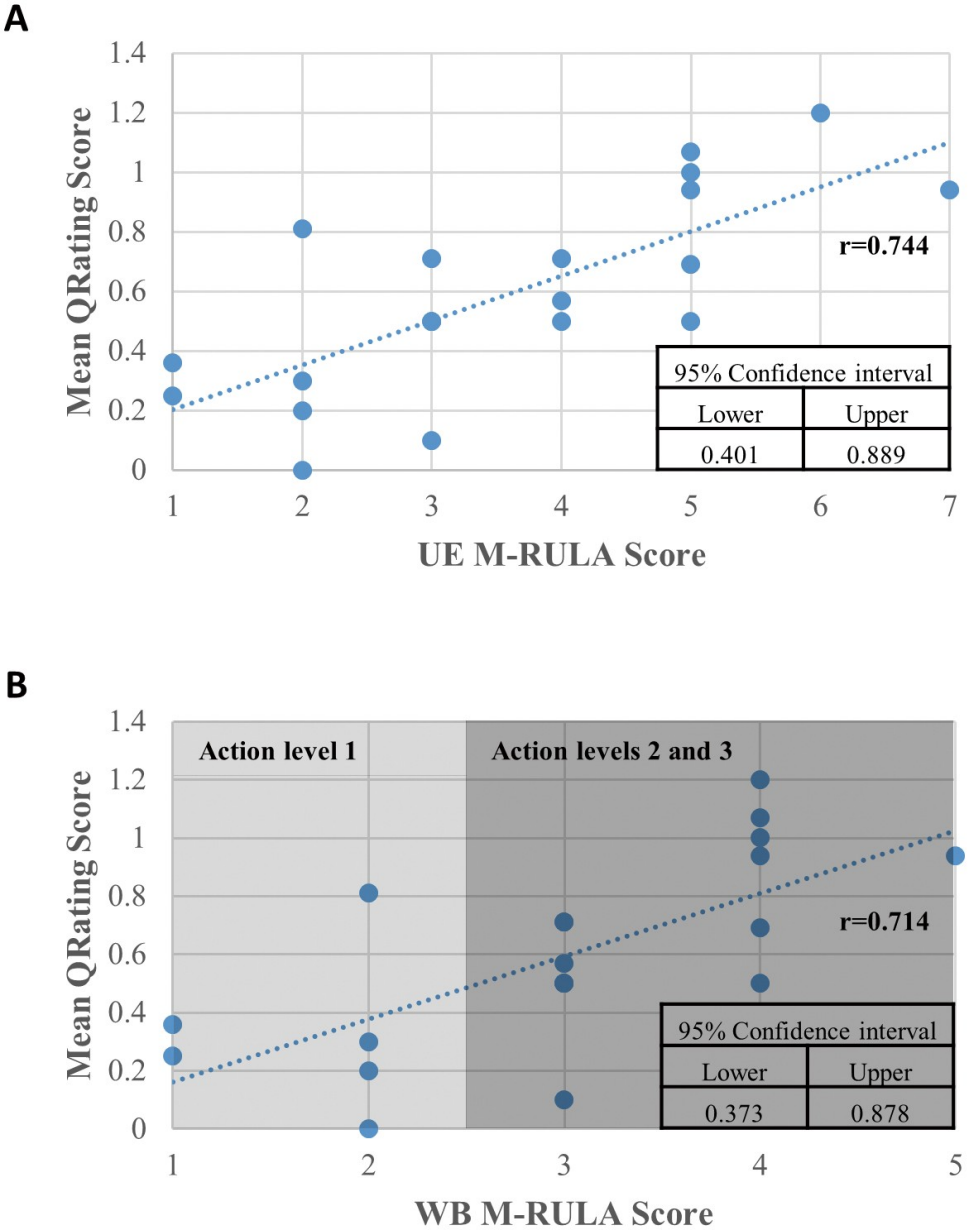
Relationship between each of the two M-RULA measures and the QRating. (A) Relationship between mean QRating and UE M-RULA scores. (B) Relationship between mean QRating and WB M-RULA scores

The relationship between each gestural discomfort measure (the QRating, the UE M-RULA and the WB M-RULA) and the baseline (the IncreaseRate) is graphically illustrated in Fig 10A–10C, along with the corresponding Spearman rank correlation coefficient. Fig 11A and 11B present the relationships between the QRating and the two M-RULA scores (the UE and the WB M-RULA scores). The relationships presented in Figs 10A–10C, 11A and 11B were all statistically significant (p-value < 0.05).

## Discussion

Figs 6–9 present the results of gestural discomfort evaluation conducted on the twenty UE gestures (Fig 5) using the QRating, the M-RULA and the IncreaseRate methods. As for the QRating method, it was found that the mean QRating scores of the twenty gestures were all low, ranging from 0 (“nothing at all”) to 1.2 (1 = “weak”) on the Borg CR10 scale (Fig 6). These low QRating values are thought to be due to the subjective discomfort rating instruction given to the participants—as described earlier, the participants were told to rate the discomfort resulting from each gesture execution trial and not to extrapolate it to some hypothetical long-term usage scenario. A small number of gesture repetitions (four times or less in the current study) would produce low physical loading, and, thus, a low perceived discomfort rating for most user-designed gestures.

The M-RULA method produced two discomfort measures: the UE and the WB M-RULA scores. The twenty gestures varied substantially in both of the M-RULA scores (Figs 7 and 8). The UE M-RULA scores of the twenty gestures ranged from 1 to 7 on the 11-point scoring system (Fig 7). The WB M-RULA scores ranged from 1 to 5 (Fig 8) on the 7-point RULA scoring system [43]. As depicted in Fig 8, out of the twenty gestures, six had the WB M-RULA scores corresponding to RULA Action level 1, thirteen for Action level 2, and one for Action level 3. According to [43], postures or motions characterized with RULA Action level 2 or higher should not be performed repeatedly or sustained over a long duration. It is interesting that fourteen out of the twenty user-designed gestures were found to be inadequate for prolonged, repetitive use. This result suggests that while the user design process may likely produce intuitive gestures [34–36,42,68,69], it does not guarantee generating comfortable or safe gestures in terms of the health of the musculoskeletal system. Thus, ergonomics gestural discomfort evaluation must be part of the gesture vocabulary design process to ensure creating low-discomfort gestures.

The mean IncreaseRate scores of the twenty gestures showed large variability ranging from 0.023 to 0.130 (Fig 9). Note that the highest IncreaseRate score was 5.6 times that of the lowest.

The main objective of the current study was to evaluate the QRating and the M-RULA methods in terms of their utility in evaluating discomfort resulting from prolonged, repetitive gesture use. This was done by comparing the mean QRating and the two M-RULA scores against the baseline mean IncreaseRate score (Fig 10A–10C).

The mean QRating and the mean IncreaseRate scores were found to be highly correlated (r = 0.838) (Fig 10A). The high agreement suggests that while the QRating method is likely to produce low rating scores for most user-designed gestures as shown in Fig 6, it can still be used to characterize and discern discomfort levels of gestures in a manner similar to the mean IncreaseRate measure—in other words, it is useful for evaluating longer-term gestural discomfort resulting from prolonged, repetitive gesture use. The previous gesture vocabulary design studies that adopted the method of subjective discomfort rating to quantify gestures’ discomfort levels may be justified on this basis [32–36,38–42].

From an information processing point of view, the high agreement between the mean QRating and the mean IncreaseRate scores (Fig 10A) implies that the human system is capable of accurately judging a gesture’s long-term discomfort level with limited information from brief physical experience. How this is accomplished is not entirely clear, but human adults may be hypothesized to have a robust and accurate internal pattern classifier constructed from prior physical experience. In occupational and daily living activities, people constantly perform various physical tasks consisting of static postures and dynamic movements, and perceive stresses/discomforts resulting from them. This would produce a large amount of motor behavior-sensory perception data accumulated over time. Such large streams of data may be used for learning high- and low-discomfort postures/movement patterns, and for constructing a robust, accurate internal pattern classifier. In this sense, most humans with enough experience might be considered as experts in the subjective evaluation of posture/movement discomfort. Such expertise—that is, an internal posture/movement classification system—would enable rapid processing of sensory data from the periphery and efference copy signals from motor commands to form reliable estimation of gestural discomfort [70–73].

Related to the human capability discussed above, it is worth pointing out that multiple previous ergonomics studies on quantifying discomfort levels of industrial postures and motions adopted subject rating protocols based on short posture holding or a small number of movement executions similar to the QRating method [24,45,74–80]. For example, in [76], the subjects rated the degree of joint discomfort only after 30 seconds of maintaining a body part in a specific position. An implicit assumption of these studies appears to be that the human information processing system can adequately characterize a manual task’s discomfort/stress level based on brief physical experience. Some of these studies demonstrated that the subjective ratings data based on short posture holding or a small number of movement executions were consistent with biomechanical or physiological measures of physical loading [24,77,79,80].

Regarding the evaluation of the M-RULA method, both the UE and the WB M-RULA scores were found to be highly correlated with the mean IncreaseRate score (r = 0.841 and 0.779, respectively) (Fig 10B and 10C). This indicates that the M-RULA scores are capable of discerning the uncomfortable from the comfortable gestures in a manner agreeing with the mean IncreaseRate score, and are useful for determining gestural discomfort resulting from prolonged, repetitive gesture use.

The usefulness of the M-RULA scores may be explained on the basis of the postural similarities shared between uncomfortable gestures and stressful conventional industrial tasks, which RULA was originally intended to detect. Visual inspection of the discomfort rankings based on the IncreaseRate score (Fig 9) revealed that the gestures with relatively high discomfort scores were associated with holding elevated and/or extended arm positions (Gestures #16, #18, #20, #19, #12, #17, #13, #14, #15 and #10). Especially, the gestures with large shoulder elevation received the highest discomfort scores (Gestures #16, #18, #19 and #20). Gestures #16, #18, #19 and #20 also involved significant wrist flexion/extension. The characteristics of high discomfort gestures observed in this study (i.e., elevated/extended arms, static posture holding and wrist flexion/extension) have been recognized as risk factors of discomfort, pain and work-related musculoskeletal disorders in many industrial ergonomics studies [79,81–86]. [31] also reported similar findings on the postural similarities between uncomfortable hand/arm gestures and stressful industrial manual tasks.

Overall, the current study demonstrated the utility of the QRating and the M-RULA methods on the basis of their observed agreement with the baseline IncreaseRate method. Both the QRating and the M-RULA methods seem practically useful as they are time- and cost-efficient. Especially, the M-RULA method does not require empirical data collection from multiple human subjects. The M-RULA method also provides information as to which aspects of a designed gesture causes the overall gestural discomfort to increase as RULA determines not only the grand score, but also the body part scores.

Related to the application of the QRating, the M-RULA and even the IncreaseRate method, one question arises: how do we determine if a designed gesture is acceptable in terms of discomfort resulting from prolonged, repetitive gesture use using each of the discomfort measures? Among the four discomfort measures (the mean QRating, the UE and the WB M-RULA and the mean IncreaseRate scores), the WB M-RULA score allows determining a gesture's acceptability/unacceptability on the basis of the four RULA Action levels [43]–as mentioned earlier, postures or motions with RULA Action levels 2 or higher are considered unacceptable for prolonged, repetitive use. As for the mean IncreaseRate score, the relationship between the mean IncreaseRate score and the WB M-RULA score depicted in Fig 10C may provide a basis for determining an acceptable limit of the mean IncreaseRate score. The criterion of the mean IncreaseRate score being less than or equal to 0.041 divides the gestures with Action level 1 and those with Action levels 2 or higher with a low misclassification rate of four out of twenty cases. For the QRating measure, the criterion of the mean QRating score being less than 0.4 divides the Action level 1 gestures from the rest with a misclassification rate of two out of twenty (Fig 11B). While more gestures need to be examined to set the acceptable/unacceptable limits for the mean QRating and IncreaseRate measures, the current study provides some initial estimates.

Some future research directions are described here: first, as demonstrated in the current study, uncomfortable gestures seem to share similarities with stressful conventional industrial manual tasks. This points to the possibility of applying various existing ergonomics evaluation techniques to gestural discomfort evaluation—in addition to RULA, other existing ergonomics work analysis tools, such as OCRA [46], Quick Exposure Check [87], and LUBA [45], may prove to be useful in evaluating discomfort levels of designed gestures. Also, biomechanical models of the upper extremities and relevant physiological measurements [88–90] may provide additional insights into gestural discomfort and may be used to further validate the QRating and the M-RULA methods. Second, the current study only dealt with discomfort evaluation of individual gestures. Further studies need to develop methods for evaluating a gesture vocabulary’s overall discomfort level. Such gesture vocabulary discomfort evaluation methods would require considering each gesture’s frequency of use and also the distribution of mechanical loading/discomfort to individual body parts.

## Conclusion

Physical discomfort during gesture-based interaction must be controlled to ensure safe and productive interaction. Controlling gestural discomfort requires an ability to adequately evaluate the discomfort levels of different gesture design alternatives.

The current study made the following methodological contributions to the measurement of gestural discomfort, and, thus, the design of safe and usable gesture-based interaction systems:

- This study, for the first time, empirically demonstrated the utility of the QRating method in evaluating designed gestures in terms of physical discomfort resulting from prolonged, repetitive gesture use, and
- This study, for the first time, proposed a gesture discomfort evaluation method based on an existing ergonomics posture evaluation tool (the M-RULA method) and demonstrated its utility in evaluating designed gestures in terms of physical discomfort resulting from prolonged, repetitive gesture use.

The two gesture evaluation methods are practically useful as they are cost- and time-efficient. They are expected to help interaction designers create safe and usable gesture-based interaction systems.

## Supporting information

S1 Table Individual dataset for the QRating measure. (DOCX)

S2 Table Individual dataset for the IncreaseRate measure. (DOCX)
