# Supplementary material for: Evaluating the utility of two gestural discomfort evaluation methods
Source: PLoS One. 2017 Apr 19;12(4):e0176123. doi: 10.1371/journal.pone.0176123 (PMC5397065; doi:10.1371/journal.pone.0176123)
Supplement: S1 Table — (DOCX) [file pone.0176123.s001.docx]

| Participant  Gesture | P1 | P2 | P3 | P4 | P5 | P6 | P7 | P8 | P9 | P10 | P11 | P12 | P13 | P14 | P15 | P16 | Mean QRating score (SD) |
| --- | --- | --- | --- | --- | --- | --- | --- | --- | --- | --- | --- | --- | --- | --- | --- | --- | --- |
| G1 | 0 | 0 | 0 | 1 | 0 | 0 | 2 | 0 | 0 | 0 | 0 | 0 | 0 | 1 | 0 | 0 | 0.3 (0.6) |
| G2 | 0 | 0 | 0 | 1 | 1 | 0 | 1 | 0 | 0 | 1 | 0 | 0 | 0 | 1 | 0 | 1 | 0.4 (0.5) |
| G3 | 0 | 0 | 0 | 0 | 0 | 0 | 0 | 0 | 0 | 0 | 0 | 0 | 0 | 0 | 0 | 0 | 0 (0) |
| G4 | 0 | 0 | 0 | 0 | 0 | 0 | 0 | 0 | 0 | 2 | 0 | 0 | 0 | 1 | 0 | 0 | 0.2 (0.5) |
| G5 | 2 | 0 | 0 | 2 | 0 | 1 | 2 | 1 | 1 | 2 | 0 | 0 | 0 | 1 | 0 | 1 | 0.8 (0.8) |
| G6 | 0 | 0 | 2 | 1 | 1 | 1 | 1 | 0 | 0 | 1 | 0 | 0 | 1 | 1 | 0 | 2 | 0.7 (0.7) |
| G7 | 0 | 0 | 0 | 0 | 0 | 0 | 0 | 0 | 0 | 1 | 0 | 0 | 0 | 1 | 0 | 0 | 0.1 (0.3) |
| G8 | 0 | 0 | 0 | 0 | 0 | 1 | 0 | 0 | 0 | 2 | 0 | 0 | 0 | 1 | 0 | 1 | 0.3 (0.6) |
| G9 | 1 | 0 | 1 | 0 | 0 | 1 | 0 | 0 | 0 | 2 | 1 | 0 | 0 | 1 | 0 | 1 | 0.5 (0.6) |
| G10 | 1 | 0 | 0 | 1 | 1 | 1 | 1 | 1 | 0 | 1 | 0 | 0 | 0 | 1 | 0 | 1 | 0.6 (0.5) |
| G11 | 1 | 0 | 1 | 0 | 0 | 1 | 1 | 0 | 0 | 1 | 0 | 1 | 0 | 1 | 0 | 1 | 0.5 (0.5) |
| G12 | 1 | 0 | 0 | 2 | 1 | 1 | 2 | 1 | 1 | 1 | 1 | 0 | 1 | 2 | 0 | 1 | 0.9 (0.7) |
| G13 | 1 | 1 | 1 | 0 | 0 | 2 | 0 | 0 | 0 | 0 | 0 | 0 | 1 | 0 | 1 | 1 | 0.5 (0.6) |
| G14 | 0 | 1 | 0 | 2 | 1 | 0 | 1 | 1 | 0 | 1 | 1 | 0 | 1 | 1 | 0 | 1 | 0.7 (0.6) |
| G15 | 1 | 0 | 0 | 1 | 1 | 1 | 1 | 1 | 0 | 1 | 0 | 0 | 1 | 1 | 0 | 2 | 0.7 (0.6) |
| G16 | 1 | 1 | 2 | 0 | 0 | 2 | 1 | 1 | 1 | 3 | 1 | 1 | 1 | 1 | 1 | 2 | 1.2 (0.8) |
| G17 | 0 | 0 | 1 | 0 | 0 | 1 | 1 | 0 | 0 | 2 | 0 | 1 | 0 | 1 | 0 | 1 | 0.5 (0.6) |
| G18 | 1 | 1 | 2 | 0 | 1 | 1 | 1 | 1 | 1 | 1 | 1 | 1 | 1 | 1 | 1 | 1 | 1.0 (0.4) |
| G19 | 1 | 0 | 0 | 2 | 1 | 1 | 2 | 1 | 1 | 1 | 1 | 1 | 1 | 1 | 1 | 2 | 1.1 (0.6) |
| G20 | 2 | 0 | 0 | 1 | 1 | 0 | 2 | 1 | 2 | 1 | 1 | 1 | 0 | 2 | 0 | 1 | 0.9 (0.8) |

**S1 Table. Individual dataset for the QRating measure**
