# Supplementary material for: Evaluating the utility of two gestural discomfort evaluation methods
Source: PLoS One. 2017 Apr 19;12(4):e0176123. doi: 10.1371/journal.pone.0176123 (PMC5397065; doi:10.1371/journal.pone.0176123)
Supplement: S2 Table — (DOCX) [file pone.0176123.s002.docx]

| Participant  Gesture | P1 | P2 | P3 | P4 | P5 | P6 | P7 | P8 | P9 | P10 | Mean number of repetitive  gesture executions (SD) | Mean IncreaseRate score (SD) |
| --- | --- | --- | --- | --- | --- | --- | --- | --- | --- | --- | --- | --- |
| G1 | 202 | 54 | 98 | 104 | 225 | 55 | 66 | 90 | 86 | 251 | 123.1 (73.8) | 0.041 (0.029) |
| G2 | 153 | 37 | 206 | 178 | 102 | 37 | 42 | 55 | 70 | 354 | 123.4 (101.9) | 0.041 (0.033) |
| G3 | 38 | 181 | 341 | 592 | 206 | 47 | 138 | 207 | 395 | 74 | 221.9 (175.3) | 0.023 (0.043) |
| G4 | 37 | 171 | 87 | 521 | 185 | 68 | 109 | 103 | 204 | 135 | 162.0 (136.8) | 0.031 (0.035) |
| G5 | 85 | 45 | 85 | 194 | 157 | 15 | 43 | 91 | 80 | 248 | 104.3 (73.2) | 0.048 (0.074) |
| G6 | 190 | 32 | 203 | 200 | 206 | 31 | 30 | 49 | 41 | 288 | 127.0 (99.0) | 0.039 (0.046) |
| G7 | 33 | 182 | 222 | 312 | 225 | 41 | 82 | 144 | 314 | 81 | 163.6 (104.4) | 0.031 (0.047) |
| G8 | 37 | 224 | 159 | 304 | 145 | 87 | 140 | 192 | 281 | 89 | 165.8 (85.8) | 0.030 (0.035) |
| G9 | 23 | 142 | 147 | 245 | 180 | 87 | 68 | 87 | 158 | 131 | 126.8 (63.1) | 0.039 (0.058) |
| G10 | 155 | 27 | 60 | 189 | 199 | 27 | 27 | 57 | 46 | 218 | 100.5 (79.6) | 0.050 (0.062) |
| G11 | 37 | 164 | 94 | 152 | 160 | 65 | 39 | 116 | 206 | 152 | 118.5 (57.9) | 0.042 (0.041) |
| G12 | 59 | 43 | 90 | 114 | 88 | 14 | 35 | 48 | 48 | 152 | 69.1 (41.6) | 0.072 (0.082) |
| G13 | 29 | 113 | 94 | 103 | 165 | 52 | 30 | 48 | 144 | 20 | 79.8 (51.3) | 0.063 (0.074) |
| G14 | 63 | 30 | 115 | 100 | 166 | 34 | 33 | 60 | 56 | 172 | 82.9 (53.1) | 0.060 (0.054) |
| G15 | 32 | 27 | 69 | 224 | 230 | 24 | 30 | 44 | 38 | 221 | 93.9 (91.4) | 0.053 (0.048) |
| G16 | 27 | 32 | 61 | 53 | 30 | 30 | 29 | 18 | 67 | 38 | 38.5 (16.2) | 0.130 (0.060) |
| G17 | 30 | 117 | 50 | 55 | 148 | 40 | 56 | 45 | 75 | 94 | 71.0 (37.8) | 0.070 (0.041) |
| G18 | 25 | 40 | 31 | 41 | 101 | 60 | 27 | 34 | 84 | 20 | 46.3 (27.0) | 0.108 (0.129) |
| G19 | 46 | 34 | 125 | 109 | 118 | 18 | 32 | 41 | 30 | 30 | 58.3 (41.7) | 0.086 (0.074) |
| G20 | 48 | 37 | 93 | 92 | 100 | 54 | 26 | 58 | 37 | 31 | 57.6 (27.7) | 0.087 (0.047) |

**S2 Table. Individual dataset for the IncreaseRate measure**
